# Shape Memory Alloys for Civil Engineering

**DOI:** 10.3390/ma16020787

**Published:** 2023-01-13

**Authors:** Cheng Fang, Canxing Qiu, Yue Zheng

**Affiliations:** 1 Department of Structural Engineering, College of Civil Engineering, Tongji University, Shanghai 200092, China; 2 Key Laboratory of Urban Security and Disaster Engineering of Ministry of Education, Beijing University of Technology, Beijing 100124, China; 3 Department of Bridge Engineering, Tongji University, Shanghai 200092, China

Shape memory alloys (SMAs) are a unique class of metals capable of recovering large strains either spontaneously or upon heating, depending on their thermal-mechanical state. Its two most common phases are the martensite and austenite phases, consisting of three different crystal structures: twinned martensite, detwinned martensite, and austenite. The prevalence of these phases is temperature dependent; the austenite phase is stable at higher temperatures and the martensite phase is stable at lower temperatures. Two different martensite crystal structures exist due to the different orientation directions (variants) of the martensitic crystal. The strain recovery properties of SMAs result from reversible phase transformations between the austenite and martensite phases, a mechanism which is attributed to shear lattice distortion rather than dislocation-based plasticity.

Since their early development in the 1960s, SMAs have been successfully applied in the medical, aerospace, robotic, and automobile industries. In the 1990s, SMA emerged as a potential material in civil engineering, and great progress in this area has been made since then. With the increasing need for more hazard resilient structural systems, the knowledge on SMAs has been widely circulated in the civil engineering community over the last two decades. A large number of relevant research projects have been granted by major funders, especially in seismically active countries. These significant investments made over the last two decades have led to considerable increases in vital knowledge that may prepare engineers for the more widespread use of SMAs in civil engineering.

However, to date, the practical application of SMAs in the construction industry has been lacking, partially due to shortcomings in engineering-oriented design approaches and a lack of effective knowledge exchanges between material scientists and civil engineers. There are still knowledge barriers across materials science and structural engineering. This Special Issue plans to present an overview of the most recent advances in the field of SMA research and its applications in civil engineering. It aims to help remove the interdisciplinary knowledge barriers and to shed considerable light on the commercialization of SMA products in the construction industry by detailing their potential applications in buildings, nuclear power plants, television transmission towers, and bridges.

Paper [1] is an element-level study that proposes a machine learning-based approach for efficient identification of thermodynamic parameters, considering the dynamic behavior of NiTi SMA wires. A feedforward artificial neural network (ANN) architecture was developed and strain rate effects were considered in a macroscopic constitutive SMA model. After training, the ANN could identify the searched model parameters from cyclic tensile stress–strain tests. The proposed approach was validated by experiments. Papers [2,3,4,5] focus on the applications of SMAs in braces and connections in residential buildings/industrial structures. Zhang et al. [2] present a novel type of hybrid self-centering braces incorporating tension-only superelastic NiTi shape memory alloy (SMA) cables and integrated viscoelastic dampers (VEDs). Their new design demonstrates an enhanced energy dissipation ability and self-centering tendency compared with existing SMA-based self-centering solutions, where its improved behavior mainly stems from the participation of the VEDs. Jia et al. [3] have developed an innovative self-centering SMA brace based on low friction slip. This study comprehensively evaluates the effects of the loading rate and initial strain on the seismic performance of the SMA braces. Both the loading rate and the initial strain are shown to have a great influence on the seismic performance of the self-centering SMA brace. They have also developed improved numerical models combined with the Graesser and Bouc–Wen models in MATLAB. Pei et al. [4] examine a new type of self-centering reinforced concrete (RC) frame beam-column joint, equipped with super-elastic SMA bars. It is shown that by using the SMA reinforcement and a steel plate, the load carrying capacity is improved, the stiffness degradation is delayed, and the ductility and self-centering ability of the joints is improved. Qian et al. [5] explore the potential of a reinforced concrete (RC) beam strengthened by SMAs and engineered cementitious composites (ECC). The test results show that, compared with ordinary reinforced concrete beams, strengthening an existing RC beam with an enlarged section area of a SMA-reinforced ECC can improve the self-recovery capacity, ductility, and deformability of the specimens.

Papers [6,7] discuss the use of SMAs in controlling the effects of seismic activity and wind on television transmission towers. Wu et al. [6] consider using SMA dampers for response reduction of a flexible television tower. A two-dimensional dynamic model was developed for dynamic computations, leading to the introduction of a mathematical model for an SMA damper. The structural dynamic responses were examined with respect to time and frequency, investigating the effects of damper stiffness, service temperature, hysteresis loops, and earthquake intensity on control efficacy. The analysis indicates that SMA dampers with optimal parameters can substantially reduce the vibrations of TV transmission towers under seismic events. Chen et al. [7] propose a wind vibration control method using SMA dampers for tower line coupled systems. Detailed parametric studies were conducted to examine the effects of the physical parameters of SMA dampers on structural responses.

With respect to bridges, Chen et al. [8] discuss a novel self-centering rocking (SCR) bridge system equipped with SMA-based piers, with a particular focus on the benefits of the SCR bridge system in the context of life cycle. Based on a life cycle loss and resilience assessment, the analysis results reveal that the novel SCR pier bridge system slightly increases the bearing displacement but extensively reduces the pier curvature ductility due to its rocking mechanism. The SCR bridge system exhibits a lower life cycle loss and exhibits a more resilient performance than a conventional bridge, especially in the regions with higher seismic intensities. Indirect loss can be significantly larger than the direct loss, specifically for earthquakes, which have a relatively low probability of occurrence. The SCR bridge system outperforms the conventional systems in terms of recovery time. Li et al. [9] examine a resilient bridge system incorporating engineered cementitious composite (ECC)-reinforced piers and SMA energy dissipation components, i.e., SMA washers. The analysis results indicate that this system has superior resilience and damage control compared to conventional bridges.

The above nine papers [1,2,3,4,5,6,7,8,9] have focussed on NiTi SMAs. Iron-based SMAs (Fe-SMAs), also known as Fe–Mn–Si alloys, are another member of the family of smart metals for civil engineering. In the civil engineering community, Fe-SMAs are well-known for their shape memory effect (SME), for which it has been widely investigated in the area of prestressing. Marinopoulou and Katakalos [10] investigate the basic thermomechanical responses of Fe-SMAs. In particular, their study focuses on the application of prestress and on the alloy’s behavior under fatigue. The effect of loading frequency on the recovery stress of the material has been thoroughly investigated. Four dog bone specimens were prepared and tested under low-cycle fatigue. Recovery stress was monitored after pre-straining and heating were applied under strain-control conditions. The measured recovery stress values are satisfactory high, verifying the prestress feasibility. In the context of earthquake engineering, Fe-SMAs are attractive due to their excellent low-cycle fatigue (LCF) resistance, in contrast to NiTi SMAs (especially large-scale SMA elements), which are brittle and exhibit poor LCF performance. This Special Issue concludes with a comprehensive review of the literature compiled by Zhang et al. [11], who provide a detailed summary of recent developments in the research and design of Fe-SMAs. The basic mechanical properties are presented and compared with conventional structural steel, and some necessary explanations are given on the metallographic transformation mechanism. Newly emerged applications such as Fe-SMA-based prestressing/strengthening techniques and seismic-resistant components/devices are discussed. It is believed that Fe-SMAs can offer a wide range of applications in the construction industry, but there are still problems that remain to be addressed and areas to be further explored. Required research on the material-, component-, and system-level is highlighted. With the systematic information provided, this work is not only of benefit to professionals and researchers who have been working in this area for a long time who want to gain an in-depth understanding of the state-of-the-art innovations in this field, but also helps enlighten a wider audience who intend to become more informed on this exciting topic.
